# Complete Genome Sequences of *Bordetella pertussis* Isolates B1917 and B1920, Representing Two Predominant Global Lineages

**DOI:** 10.1128/genomeA.01301-14

**Published:** 2014-12-24

**Authors:** Marieke J. Bart, Anne Zeddeman, Han G. J. van der Heide, Kees Heuvelman, Marjolein van Gent, Frits R. Mooi

**Affiliations:** a Laboratory of Pediatric Infectious Diseases, Department of Pediatrics, Radboud University Medical Center, Nijmegen, The Netherlands; b Centre for Infectious Diseases Control (CIb), National Institute of Public Health and the Environment (RIVM), Bilthoven, The Netherlands

## Abstract

*Bordetella pertussis* is the causative agent of pertussis, a disease which has resurged despite vaccination. We report the complete, annotated genomes of isolates B1917 and B1920, representing two lineages predominating globally in the last 50 years. The B1917 lineage has been associated with the resurgence of pertussis in the 1990s.

## GENOME ANNOUNCEMENT

*Bordetella pertussis* is the main causative agent of whooping cough, or pertussis, a disease of the respiratory tract that is especially severe for infants. It is estimated that in 2010 pertussis resulted in 81,400 (CI 3,000 to 399,000) deaths, mainly in children (1). In the 1990s a resurgence of pertussis was observed in many vaccinated populations. Several causes may have contributed to this phenomenon, including increased awareness, low vaccine coverage, waning immunity, the switch from whole-cell vaccines to acellular vaccines, and pathogen adaptation (2). Studies of *B. pertussis* populations have identified two lineages that have predominated in the last 50 years, characterized by the alleles for the pertussis toxin promoter (*ptxP*) and the pertussis toxin A subunit (*ptxA*) (3–6). Strains with the genotype *ptxP1*-*ptxA1*, predominated in the period 1960–1990. These strains were replaced by strains with the genotype *ptxP3*-*ptxA1* in the 1990s, in a global selective sweep. Here we present the complete, closed, and annotated genome sequences of the isolates B1920 and B1917, which are representatives of the *ptxP1*-*ptxA1* and *ptxP3*-*ptxA1* lineages, respectively. Both strains were isolated from Dutch patients in 2000. Unannotated contigs of these strains, generated by 454 reads, were submitted previously (7). We believe that the B1917 and B1920 strains are more suitable reference strains than the Tohama I strain, which is now widely used (7). The Tohama I strain has been subcultured since the 1950s and is phylogenetically relatively distant from currently circulating strains (8).

Strains were grown on Bordet-Gengou agar supplemented with 15% sheep’s blood and incubated for 3 days at 35°C. Genomic DNA was isolated using a QIAGEN Genomic-tip 100/G kit (Qiagen, Hilden, Germany) according to the manufacturer’s instructions for Gram-negative bacteria, and a 10-kb library was prepared. Sequencing was performed using PacBio RS system using 6 SMRT cells per genome. The generated sequences were *de novo* assembled with HGAP (9) and trimmed and rotated by hand, resulting in a single, circular contig for both genomes. *B. pertussis* genomes are highly similar and therefore RATT (10) was used to transfer annotations from the *B. pertussis* reference genomes Tohama I, CS, and 18323 (8, 11). Subsequently, sequenced genomes were manually checked for genes not present in the reference genomes. The complete genome of *B. pertussis* B1917 comprises 4,102,186 bp and contains 3,820 genes, including 361 pseudogenes and 277 transposase genes. The complete genome of *B. pertussis* B1920 comprises 4,114,630 bp and harbors 3,827 genes, including 365 pseudogenes and 284 transposase genes. Both genomes contain 51 tRNA genes and 3 rRNA operons. A comparison of both genomes revealed three large inversions, a large deletion of 19 and 16 genes from *B. pertussis* B1917 and B1920, respectively, 29 small insertion or deletion events, and 223 single nucleotide polymorphisms.

### Nucleotide sequence accession numbers.

The whole-genome sequences have been deposited in DDBJ/ENA/GenBank under the accession numbers CP009751 and CP009752 for *B. pertussis* B1917 and B1920, respectively. The versions described in this paper are the first versions.
